# Supplementary material for: The transformation of sensory to perceptual braille letter representations in the visually deprived brain
Source: eLife. 2024 Dec 4;13:RP98148. doi: 10.7554/eLife.98148 (PMC11616995; doi:10.7554/eLife.98148)
Supplement: Supplementary file 1. [file elife-98148-supp1.docx]

| **Experiment** | **ID** | **Sex** | **Age** | **Blindness onset** | **Etiology** | **Light perception** |
| --- | --- | --- | --- | --- | --- | --- |
| fMRI | EB01 | F | 24 | birth | Cataract | yes |
| fMRI | EB02 | M | 35 | birth | Retinopathy of prematurity | yes |
| fMRI | EB03 | M | 36 | 2 years | Retinoblastoma | no |
| fMRI | EB04 | M | 39 | 3 years | Retinoblastoma | no |
| fMRI | EB05 | F | 33 | birth | Retinopathy of prematurity | yes |
| fMRI | EB06 | M | 36 | birth | Unknown hereditary disease | no |
| fMRI | EB07 | F | 53 | birth | Glaucoma | yes |
| fMRI | EB08 | F | 34 | birth | Optic nerve atrophy | yes |
| fMRI | EB09 | F | 30 | birth | Unknown malformation | no |
| fMRI | EB10 | F | 47 | birth | Retinopathy of prematurity | no |
| fMRI, EEG | EB11 | M | 55 | birth | Retinopathy of prematurity | no |
| fMRI, EEG | EB12 | F | 42 | birth | Tapetoretinal degeneration | yes |
| fMRI, EEG | EB13 | M | 55 | birth | Retinitis Pigmentosa | no |
| fMRI, EEG | EB14 | F | 38 | birth | Leber Congenital Amaurosis | yes |
| fMRI, EEG | EB15 | F | 35 | birth | Optic nerve atrophy | yes |
| EEG | EB16 | M | 48 | birth | Retinopathy of prematurity | no |
| EEG | EB17 | F | 61 | 3 years | Cataract | no |
| EEG | EB18 | F | 42 | birth | Retinopathy of prematurity | no |
| EEG | EB19 | F | 29 | birth | Leber Congenital Amaurosis | yes |
| EEG | EB20 | F | 41 | birth | Retinopathy of prematurity | no |
| EEG | EB21 | F | 55 | birth | Retinopathy of prematurity | no |
